# The transfer of specific mitochondrial lipids and proteins to lipid droplets contributes to proteostasis upon stress and aging in the eukaryotic model system *Saccharomyces cerevisiae*

**DOI:** 10.1007/s11357-019-00103-0

**Published:** 2019-11-01

**Authors:** Florian Geltinger, Julia Tevini, Peter Briza, Amrito Geiser, Johannes Bischof, Klaus Richter, Thomas Felder, Mark Rinnerthaler

**Affiliations:** 1 Department of Biosciences, University of Salzburg, Salzburg, Austria; 2 Department of Laboratory Medicine, Paracelsus Medical University, Salzburg, Austria; 3 Obesity Research Unit, Paracelsus Medical University, Salzburg, Austria

**Keywords:** Replicative aging, Mitochondria, Lipid droplets, Protein sink, Proteome, Lipidome

## Abstract

Originally Lipid droplets (LDs) were considered as being droplets for lipid storage only. Increasing evidence, however, demonstrates that LDs fulfill a pleiotropy of additional functions. Among them is the modulation of protein as well as lipid homeostasis. Under unfavorable pro-oxidative conditions, proteins can form aggregates which may exceed the overall proteolytic capacity of the proteasome. After stress termination LDs can adjust and support the removal of these aggregates. Additionally, LDs interact with mitochondria, specifically take over certain proteins and thus prevent apoptosis. LDs, which are loaded with these harmful proteins, are subsequently eliminated via lipophagy. Recently it was demonstrated that this autophagic process is a modulator of longevity. LDs do not only eliminate potentially dangerous proteins, but they are also able to prevent lipotoxicity by storing specific lipids. In the present study we used the model organism *Saccharomyces cerevisiae* to compare the proteome as well as lipidome of mitochondria and LDs under different conditions: replicative aging, stress and apoptosis. In this context we found an accumulation of proteins at LDs, supporting the role of LDs in proteostasis. Additionally, the composition of main lipid classes such as phosphatidylcholines, phosphatidylethanolamines, phosphatidylinositols, phosphatidylglycerols, triacylglycerols, ceramides, phosphatidic acids and ergosterol of LDs and mitochondria changed during stress conditions and aging.

**Electronic supplementary material:**

The online version of this article (10.1007/s11357-019-00103-0) contains supplementary material, which is available to authorized users.

## Introduction

Among many theories of aging the most prominent is the “Free Radical Theory of Aging” (FRTA) which is probably the most cited theory in this field. This theory was proposed in the year 1956 by Denham Harman (Harman 1956), who suggested that so called reactive oxygen species (ROS) dramatically increase over lifetime. ROS then may react with many organic components of the cell including DNA, proteins and lipids, causing cellular damage limiting the lifespan of most organisms. On the one hand, many examples exist, in which a dramatic increase in ROS levels in fact shortens the lifespan of several organisms. On the other hand, an only moderate increase in ROS levels can even extend lifespan, whereas high doses of anti-oxidants can actually decrease life expectancy (Davies et al. 2017; Lapointe and Hekimi 2010; Perez et al. 2009; Stuart et al. 2014). Therefore, the overall picture clearly needs some adjustment and a more specific case-to-case assessment. A possible explanation for this apparent difficulty lies in the fact that especially hydrogen peroxide can act as second messenger and thus fulfills important functions in the cell (Rinnerthaler et al. 2012; Sies 2014). If oxidative damage exceeds a certain threshold and ROS levels overcharge the cellular anti-oxidative defense systems, apoptosis is induced. In fact, induction of apoptosis is one of the hallmarks of aging (Laun et al. 2007; Laun et al. 2001; Lopez-Otin et al. 2013). The apoptotic program takes place stepwise and initiates a shuttling of particular proteins to mitochondria. In mammals the number of these proteins is high and includes MCL1, BMF, BAX, BCL2, BCL-XL, NOXA, Puma, BAD, BAK, BID, BIK and BIM (Happo et al. 2012). In yeast cells, similar proteins include Ybh3 and Mmi1 among many others (Braun et al. 2006; Buttner et al. 2011). Some of these proteins induce changes in and at mitochondria that include fragmentation of the mitochondrial network, mitochondrial outer membrane permeabilization, calcium influx and cyctochrome c release. The apoptotic program is a highly regulated process and many steps are reversible at early stages. Bcl-XL blocks the permeabilization of the mitochondrial outer membrane, which is initiated by the oligomerization of BAX and BAK (Dlugosz et al. 2006). This oligomerization is a prerequisite for cytochrome c release from mitochondria which initiates a caspase cascade that can be blocked by IAPs (inhibitor of apoptosis proteins) (Yang and Li 2000).

In any case, dysfunctional mitochondria with an altered protein as well as lipid composition develop. A specialized form of autophagy, termed mitophagy facilitates the removal of these mitochondria. In fact there is a complex crosstalk between autophagy and apoptosis (Bitto et al. 2014; Fan and Zong 2013). Damaged mitochondria are identified by mitochondrial network fragmentation and a collapse of the mitochondrial membrane potential. Subsequently, these mitochondria are engulfed in autophagosomes, transported to lysosomes/vacuoles and are degraded finally (Shirihai et al. 2015).

In a recent publication we identified an alternative route to detoxify affected mitochondria (Bischof et al. 2017). The alternative pathway utilizes lipid droplets (LDs), which are nowadays discussed as distinct cellular organelles. LDs are composed of a neutral lipid rich core (triacylglycerols as well as sterol esters) surrounded by a unique phospholipid monolayer. Different kinds of proteins are able to attach or even integrate into this monolayer.

We recently demonstrated that the number of LDs increases within the cell upon stress and apoptosis. Furthermore, these LDs appear to get in close contact with mitochondria. It was demonstrated that fragmentation of the mitochondrial network increases the LD-mitochondria interaction (Bischof et al. 2017; Shaw et al. 2008; Wang et al. 2011). The LD-mitochondria crosstalk includes the formation of a lipid bridge that is eventually stabilized by a protein clamp (Bohnert 2018). The high LD surface tension supports the removal of proteins from the outer mitochondrial membrane. We identified several proteins that shuttle between these two organelles: Erg6p and Mmi1p in yeast cells and BAX and Bcl-XL in mammalian liver cells (Bischof et al. 2017). LDs can supply lipids to mitochondria, which are essential for beta-oxidation and thus energy production (Welte and Gould 2017). It seems also likely that some toxic lipid products are removed from mitochondria in such a way to prevent lipotoxicity. Due to this exchange, the LDs themselves may accumulate toxic molecules, becoming potentially dangerous particles for the cell. Therefore LDs are finally degraded in the vacuole/lysosome via the specialized autophagic mechanism lipophagy (van Zutphen et al. 2014). The released neutral lipids are available for energy production and promote cell growth after “stressful” conditions.

During stress conditions not only the protein profiles of LDs and mitochondria change, but also does the lipid composition. In this work, we analyzed several conditions (replicative aging, proteotoxic stress and apoptosis) and how they impact the proteome and lipidome of mitochondria and LDs. Additionally, we aimed to demonstrate that various proteins shuttle from mitochondria to LDs upon aging and apoptosis. Moreover, we detected dramatic changes in the lipidome of both organelles in response to the tested conditions.

## Materials and methods

### Yeast strains

For this study the *S. cerevisiae* strain BY4741 (MATa his3Δ1 leu2Δ0 met15Δ0 ura3Δ0) was used. The LD deficient quadruple deletion mutant strain (QM) (*Δare1, Δare2, Δdga1, Δlro1*) was created previously (Bischof et al. 2017). Additionally the yeast strain BY4741 ERG13::GFP::HIS3MX6 with a GFP tagged open reading frame of the Huh-collection was used (Huh et al. 2003). In either complex medium (YPD or YPGal) (1% (*w*/*v*) yeast extract, 2% (w/v) peptone and 2% (w/v) D-glucose or supplemented with D-galactose) or in Synthetic complete glucose medium (SC-glucose) (2% (w/v) d-glucose, 0.17% (w/v) yeast nitrogen base without amino acids supplemented with 0.5% ammonium sulphate and 10 ml of complete dropout mixture (0.2% Arg, 0.1% His, 0.6% Ile, 0.6% Leu, 0.4% Lys, 0.1% Met, 0.6% Phe, 0.5% Thr, 0.4% Trp, 0.1% Ade, 0.4% Ura, 0.5% Tyr) per liter)) the yeast strains were grown at 28 °C.

### Elutriation

The elutriation was performed as described earlier (Streubel et al. 2018). At 28 °C overnight cultures of BY4741 and the LD deficient QM strain were grown. The respective culture was then diluted to an OD_600_ of 0.1 in 400 mL YPD medium and grown for one day until stationary phase. We used the standard elutriation chamber of the JE-6B rotor of the Beckman elutriation system. The elutriation system requires single yeast cells, therefore daughter cells were separated from their mother cells via sonication. Initially cells were pelleted at 3500 rpm for 5 min and washed once with 50 mL of PBS buffer before re-centrifugation. The remaining pellet was resuspended in 10 mL PBS buffer before sonification. Afterwards cells were loaded into the elutriation chamber and elutriated at a rotor speed of 2700 rpm with a flow rate of 10 mL/min. The rotor speed was reduced to 1350 rpm to collect fractions III-V (middle-aged and old cells), which were re-inoculated into YPD medium to increase the yield of cells at higher age. A second elutriation round with altered settings was performed after 2 days of growth. The cells were loaded at a rotor speed of 3200 rpm and a reduction of it to 2700 rpm yielded fraction II (young cells). To collect fraction V cells (old cells), the rotor speed was reduced to 2000 rpm stepwise to remove middle aged cells (fraction II and IV). At a rotor speed of 1350 rpm fraction V cells were collected.

### Isolation of mitochondria

Mitochondria were isolated from young and old cells obtained from elutriation. Additionally cells of the following strains were used: BY4741 pCM66, BY4741 pCM666 BAX, QM pCM666 and QM pCM666 BAX. These strains were inoculated to an OD_600_ of 0.1 and grown for 24 h in SC-Leu medium. After 12 h of growth doxycycline was added to yield a concentration of 200 mg/L and cells were grown for another 12 h, to induce BAX expression. Cells were pelleted for 5 min at 3000 rpm. The pellet was resuspended in 2 ml ice-cold Sorbitol B buffer (0.7 M sorbitol, 50 mM Tris pH 7.5, 0.2 mM EDTA). The cells were broken with glass beads (diameter 0.25–0.5 mm) in a bead beater (MP Biomedicals FastPrep −24 Classic Instrument) for 30 s with 4500 movements/s. The suspension was centrifuged at 800 x g for 5 min at 4 °C and the supernatant pelleted at 15.000 x g for 15 min and dissolved in 1.5 ml Sorbitol B buffer. These two alternating centrifugation steps (800 x g for 5 min and 15.000 x g for 15 min) were repeated four times. With the last centrifugation step at 15.000 x g purified mitochondria were obtained for further analyses.

### Lipid droplet isolation

For the LD isolation, the same strains and culture conditions were used (for obvious reasons LDs were not isolated from the QM strains). Cells were washed and resuspended in 20 ml of isolation buffer 1 (0.1 M Tris-HCl, 10 mM DTT pH 9.4) and incubated at 28 °C for 15 min. Cells were washed, pelleted and resuspended in 25 ml isolation buffer 2 (1.2 M Sorbitol, 20 mM tri-potassium phosphate pH 7.4). For subsequent cell wall digestion the isolation buffer 2 was supplemented with 0.5 mg/ml zymolyase and the resuspended cells were incubated for 1 h at 28 °C. Next, cells were broken with 20 strokes in a Potter homogenizer and centrifuged for 30 min at 29.000 rpm (100.000 x *g*) in an ultracentrifuge. Afterwards the crude LD fraction floating on top was collected with the use of a special self-made suction device. To remove cytosolic contaminations a second ultracentrifugation step was performed for 30 min at 29.000 rpm (100.000 x *g*). To this end, the sample was mixed with sucrose and sodium carbonate (final concentrations 25 and 10 mM, respectively). This mixture was pipetted on top of a 60% sucrose cushion and overlaid with 10 mM sodium carbonate. The top layer of this gradient consisted of 200 mM Tris pH 7.4 buffer. After re-centrifugation (30 min at 100.000 x g), the purified LDs were collected.

### Protein identification in mitochondria and delipidated LDs

For protein identification, mitochondria were suspended in Sorbitol B buffer to a final volume of 100 μl. LDs were mixed with 100 μl ice cold acetone and vortexed thoroughly. After overnight precipitation at −25 °C, the samples were centrifuged at 20.000 g for 15 min. The supernatant was discarded and the pellet washed thoroughly with acetone. After acetone evaporation, the pellet was dissolved in 100 μl of PBS buffer. Prior to tryptic digests, proteins in the LD samples were precipitated by adding 25 μl 100% (*w*/*v*) trichloroacetic acid. The precipitate was washed with acetone, dried and resuspended in digestion buffer.

Samples were reduced, alkylated and digested with the ProteoExtract All-in-One Trypsin Digestion Kit (Merck Millipore). After desalting with ZipTip C18 (EMD Millipore), peptides were loaded on a Acclaim PepMap RSLC column (C18, 75 μm × 15 cm) and the column was developed with an acetonitrile gradient (solvent A: 0.1% (*v*/v) formic acid; solvent B: 0.1% (v/v) formic acid/90% (v/v) acetonitrile; 5–45% B in 140 min) at a flow rate of 300 nl/min at 55 °C. The HPLC (Dionex Ultimate 3000, Thermo Fisher Scientific) was directly coupled via nano-electrospray to a Q Exactive Orbitrap mass spectrometer (Thermo Fisher Scientific). Capillary voltage was 2 kV. For peptide identification, a top 12 method was used, with the normalized fragmentation energy at 27%. Proteins were identified with PEAKS Studio X (Bioinformatics Solutions), using the *Saccharomyces cerevisiae* ATCC204508 proteome data from UniProt. Quantification was done label-free and is based on peptide signal intensities.

### Lipid identification in mitochondria and LDs

For lipid identification, LDs were adjusted to OD_600_ of 0.1 with 200 mM Tris pH 7.4 buffer. Mitochondria were similarly adjusted with Sorbitol B buffer. LDs and mitochondria were stored at −80 °C until sample preparation according to a modified Bligh&Dyer protocol (Pellegrino et al. 2014). Before extraction, 10 μL of a synthetic lipid standard mastermix (including 15 deuterated lipids) were added to 90 μL of extraction buffer containing lipid droplets or mitochondria. Lipid extracts were analyzed by flow injection analysis (FIA) shotgun lipidomics using an ekspert MicroLC 200 system (eskigent, Singapore) connected to a TripleTOF 4600 System (AB SCIEX, Darmstadt, Germany) as reported earlier (Simons et al. 2012). Each sample was injected twice, for one measurement in positive and one for negative ionization mode, respectively. We used Analyst® TF Software (v1.7, AB SCIEX, Darmstadt, Germany) for instrumental controlling and data acquisition. Data were processed with Lipid View™ software (v1.3 beta, AB SCIEX, Darmstadt, Germany) and lipid identification was based on high-resolution precursor ion and neutral loss scans specific for proposed lipid species. Internal standard correction for each lipid was carried out by normalization against the appropriate synthetic isotopically labeled lipid standard purchased from Avanti Lipids (18:1 (d7) MAG, 791646C; 15:0–18:1 (d7) DAG, 791647C; 15:0–18:1 (d7)-15:0 TAG, 791648C; 15:0–18:1 (d7) PC, 791637C; 15:0–18:1 (d7) PE, 791638C; 15:0–18:1 (d7) PS, 791639C; 15:0–18:1 (d7) PG, 791640C; 15:0–18:1 (d7) PI, 791641C; 15:0–18:1 (d7) PA, 791642C; 18:1 (d7) LPC, 791644C; 18:1 (d7) Cholesteryl Ester, 111,015; 18:1 (d7) Sphingomyelin, 791649C; 16:0 (d31) Ceramide, 868516P; C15 Ceramide (d7), 860681P; Sphingosine (d7), 860657P).

### Nile red, DAPI and DASPMI staining

Yeast strains were grown in YPGal to mid-exponential phase and washed twice with PBS. Finally, cells were resuspended in PBS containing 0.01 mg/ml Nile red (Thermo Fisher Scientific; N1142) and incubated for 15 min in the dark. Afterwards cells were used for fluorescence imaging. Prior to DAPI staining yeast cells were washed twice in PBS followed by ethanol permeabilization (in 100% EtOH) for 3 min. Cells were pelleted and resuspended in 200 μl PBS at a final concentration of 300 nM DAPI (Thermo Fisher Scientific; D3571). After 5 min of incubation, cells were washed with PBS for imaging. For the colocalization with mitochondria cells were washed twice. The remaining pellet was dissolved in 500 μl of a 5 μM suspension of DASPMI (Thermo Fisher Scientific; D288) in PBS. Cells were then incubated at 28 °C under constant shaking in the dark. Before fluorescence microscopy cells were washed twice with PBS.

### Fluorescence microscopy

For the imaging of the GFP fusion proteins, Nile red, DASPMI and DAPI staining a 100x Plan Apochromat objective (NA = 1.4) by Nikon (Tokyo, Japan) connected to an Eclipse Ni-U microscope equipped with a DS-Fi2 digital camera was used in combination with the Nikon NIS-Elements Ar imaging software. The filter blocks DAPI and TRITC were used for co-localization with DAPI, Nile red and DASPMI. Additionally for the detection of GFP a Nikon GFP-L filter block (excitation 460–500 nm; emission >510 nm) was used.

### ImmunoBlot

LDs used for ImmunoBlot analysis were equilibrated at the same OD_600_ and equal amounts of material were loaded. Samples were mixed with the respective sample buffer, loaded onto a 13.5% SDS-PAGE gel and afterwards blotted on a protran BA85 nitrocellulose membrane (Schleicher & Schuell BioScience GmbH, Dassel, Germany) (250 mA, 90 min at RT). The membrane was blocked with MTBS-T (25 mM TRIS pH 7.6, 137 mM NaCl; 0.1% TWEEN 20, 5% nonfat milkpowder) for 90 min at RT. After washing for 30 min in TBS-T the primary antibody (GFP-antibody (B-2) HRP (sc-9996; Santa Cruz)) was diluted 1:1000. The membrane was incubated at 4 °C overnight and then washed three times for 10 min each with TBS-T, before the incubation with the secondary antibody. A dilution of 1:25.000 in MTBS-T (5% *w*/*v* milk powder in TBS-T) of the polyclonal rabbit anti-mouse immunoglobulins/HRP; P0161; Dako was prepared. After three additional washing steps with TBS-T the Pierce ECL western blotting substrate (Thermo Fischer Scientific, Waltham, MA, USA) according to the manufacturer’s instructions was used for chemiluminescence detection.

### Statistical analysis

Data are presented as arithmetic means ± S.E.M. We used a two-tailed Student’s *t* test and results with a *p* < 0.05 were considered as statistically significant.

## Results and discussion

In a previous publication (Bischof et al. 2017) we could demonstrate that LDs have a function as stress sensors, reacting to harmful conditions in several ways. An immediate response was an increase of LD biomass indicating that LDs change their lipid composition upon stress. Additionally, we demonstrated that specific mitochondrial proteins such as mammalian BAX and BCL-XL, as well as Mmi1 (Bischof et al. 2017) and Erg6 in yeast cells are transferred from mitochondria to LDs. The removal of these proteins from the outer mitochondrial membrane (OMM) constitutes a pro-survival signal and increases the fitness of yeast as well as mammalian cells. Currently only scarce knowledge is available on the number of proteins that can shuttle from mitochondria to LDs. Therefore, we decided to study the proteome of stressed and unstressed LDs in detail.

We used the ectopic expression of mammalian BAX in yeast cells as the main stressor for various reasons. (1) Expression of the murine BAX protein exhibits a cytotoxic effect on yeast cells (Kissova et al. 2000); (2) Cytotoxicity seems to be focused on mitochondria (Kissova et al. 2000); (3) Expression of BAX increases the cellular LD content (Bischof et al. 2017). Therefore, yeast cells were either transformed with a plasmid containing mBAX under control of a doxycycline inducible promoter (pCM666-mBAX) or the empty control vector (pCM666).

**Fig. 1:**
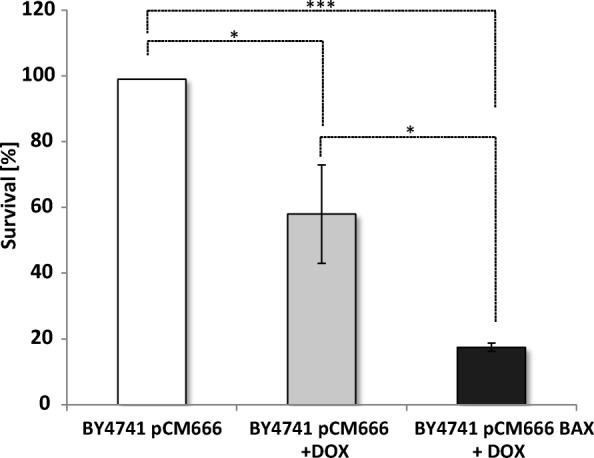
**Survival upon BAX expression**. The strains BY4741 pCM666 and BY4741 pCM666-mBAX were analyzed. BAX expression as induced by the addition of 200 mg/l doxycycline. Expression of BAX clearly reduces cell viability. The values represent the mean ± S.E.M. of 3 biological replicates, the significance of difference was analyzed by Student’s *t* test (*: *p* < 0.1; **: *p* < 0.05; ***: *p* < 0.01)

As shown in Fig. 1, the addition of doxycycline itself decreased cell survival due to proteotoxic stress at mitochondria (Moullan et al. 2015). A well-established way to induce apoptosis in yeast cells is the heterologous expression of BAX (Carmona-Gutierrez et al. 2018). As expected, the doxycycline-induced expression of BAX further promoted cell death (approximately 20% cell survival in contrast to 60% cell survival was observed). Based on these findings, we isolated LDs from cells treated in three different ways: (1) Unstressed, (2) mildly stressed (BY4741 pCM666 + 200 mg/l doxycycline) and (3) heavily stressed/apoptotic cells (BY4741 pCM666-mBAX +200 mg/l doxycycline; expression established in (Bischof et al. 2017)). As the aging process of yeast (either replicative or chronological) is closely related to apoptosis (Laun et al. 2012), we analyzed the LDs of replicatively aged yeast cells too.

### The age and stress proteome of LDs

**Fig. 2:**
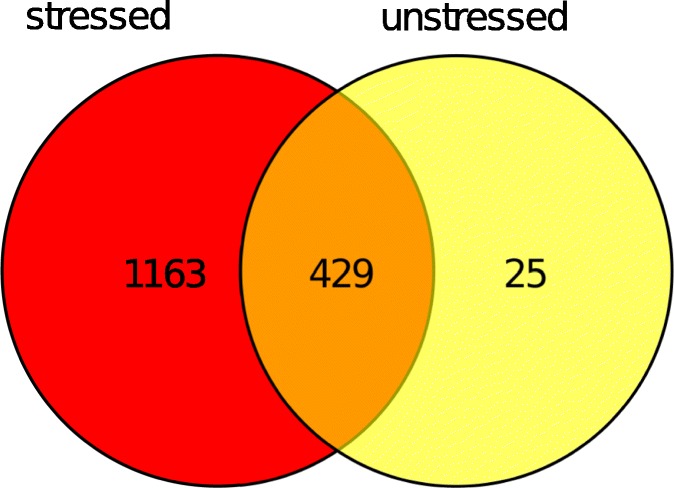
**Venn diagram** comparing the LD proteomes isolated from unstressed and mildly stressed cells (200 mg/L doxycycline). Numbers indicate identified proteins

In a first approach, cells of the BY4741 as well as the LD deficient QM strain were cultured each one either harboring pCM666 or pCM666 BAX. Cells were stressed for 12 h by the addition of doxycycline inducing BAX expression. We then isolated pure LDs, avoiding cytosolic contaminations, in triplicates and extracted proteins for subsequent mass spectrometry analysis. MS analyses revealed that even in unstressed cells the number of proteins at LDs differed (215–454 proteins were identified in the triplicates) between strains. Our findings confirm published literature reporting MS analysis on yeast LDs, which report 76 to 440 identified proteins in LDs (Currie et al. 2014; Fei et al. 2011b; Grillitsch et al. 2011). The variance in protein content may be caused by the fact that LDs never lose contact with the endoplasmic reticulum. Therefore, proteins and lipids are constantly exchanged via a lipidic bridge over the entire LD lifetime (Bohnert 2018), precluding a distinct LD proteome. Additionally, LDs can detach from their place of origin and can get in close physical contact with several target organelles by protein clamps or lipidic bridges (Schuldiner and Bohnert 2017). Such target organelles include mitochondria, peroxisomes, the nucleus, the Golgi apparatus and the vacuole (Gao and Goodman 2015). Consequently, it is expected that MS analysis also detects co-purified proteins of these interconnected organelles (Schmidt et al. 2013). Additionally, LDs can vary greatly in size (small, middle sized and large LDs can be distinguished), and these LD subpopulations are distinct in their protein composition (Fei et al. 2011b; Zhang et al. 2016). We compared our protein dataset from unstressed LDs with the proteome of reported wild type yeast LDs (Fei et al. 2011b), and found an overlap of more than 60%. As discussed above, our results clearly support the finding that there is no characteristic proteome of LDs in yeast, but the LD proteome varies a lot under different conditions. Various factors including growth condition, growth phase, age and applied stressors influence the proteome as well as the lipidome of LDs. Accordingly the application of doxycycline alone induced changes in the lipid and protein composition of LDs in our experiments. The application of mild proteotoxic stress (addition of doxycycline) and apoptosis (heterologous BAX expression) induced a dramatic increase in protein content (Fig. 2). The core proteome remained unchanged (429 proteins), but some 1000 proteins additionally localized to LDs under stress conditions. The increase in protein numbers did not depend on the applied stress (either doxycycline treatment or BAX expression), and the proteins identified at the LDs overlap to a large extend (approximately 88% overlap). Overall 702 proteins localized to LDs in response to stress induction and apoptosis. We clustered these proteins according to their predicted localization using the David Bioinformatics database (https://david.ncifcrf.gov/). By this approach we did not only identify typical LD proteins, as 17% of all proteins were of cytosolic origin, 31% were nuclear, 18% mitochondrial, 20% ER specific, 8% from the Golgi apparatus, 4% from the vacuole and 1.3% peroxisomal. Furthermore it is well described that many different stresses can result in oxidative modifications of proteins, initiating protein unfolding and crosslinking. A common finding is the clustering of these modified proteins into aggregates (Rinnerthaler et al. 2015). Different intracellular protein aggregates with distinct cellular localization were described previously: JUNQ (juxtanuclear quality control compartment, IPOD (insoluble protein deposit) and inclusion bodies (IBs) (Escusa-Toret et al. 2013; Kaganovich et al. 2008; Plafker 2010). IBs are of special interest, as they partially co-localize with LDs. Furthermore, it was demonstrated that LDs are a pre-requisite for efficient IB clearing and thus contribute to proteostasis (Moldavski et al. 2015).

We recently demonstrated that selected mitochondrial proteins are shuttled from mitochondria to LDs (Bischof et al. 2017), and that removal of these proteins from the OMM significantly improves cellular fitness and cell survival.

In the present work, we clearly demonstrate that proteins accumulate at LDs in stressed cells (either mild stress or apoptosis). This finding supports a role of LDs as protein sink for potentially harmful proteins. Additionally we aimed to analyze whether the stressor “replicative aging” induces a similar accumulation of proteins at LDs. Replicative aging in yeast is recognized as the ability of mother cells to produce a limited amount of mitotic daughter cells. This differs from chronological aging, which describes the survival of yeast cells in stationary phase after a prolonged period of time. Replicative aging is accompanied with an increase in the intracellular appearance of several apoptotic markers, mostly mitochondrial ROS, and an aggregation of proteins (Laun et al. 2007; Laun et al. 2001). Therefore, it seemed possible that LDs accumulate proteins in aged cells. We therefore isolated old BY4741 and quadruple mutant strain yeast cells via elutriation, a special kind of counterflow centrifugation. This technique utilizes the dramatic increase in cell size and sedimentation coefficient of replicatively aged cells.

**Fig. 3:**
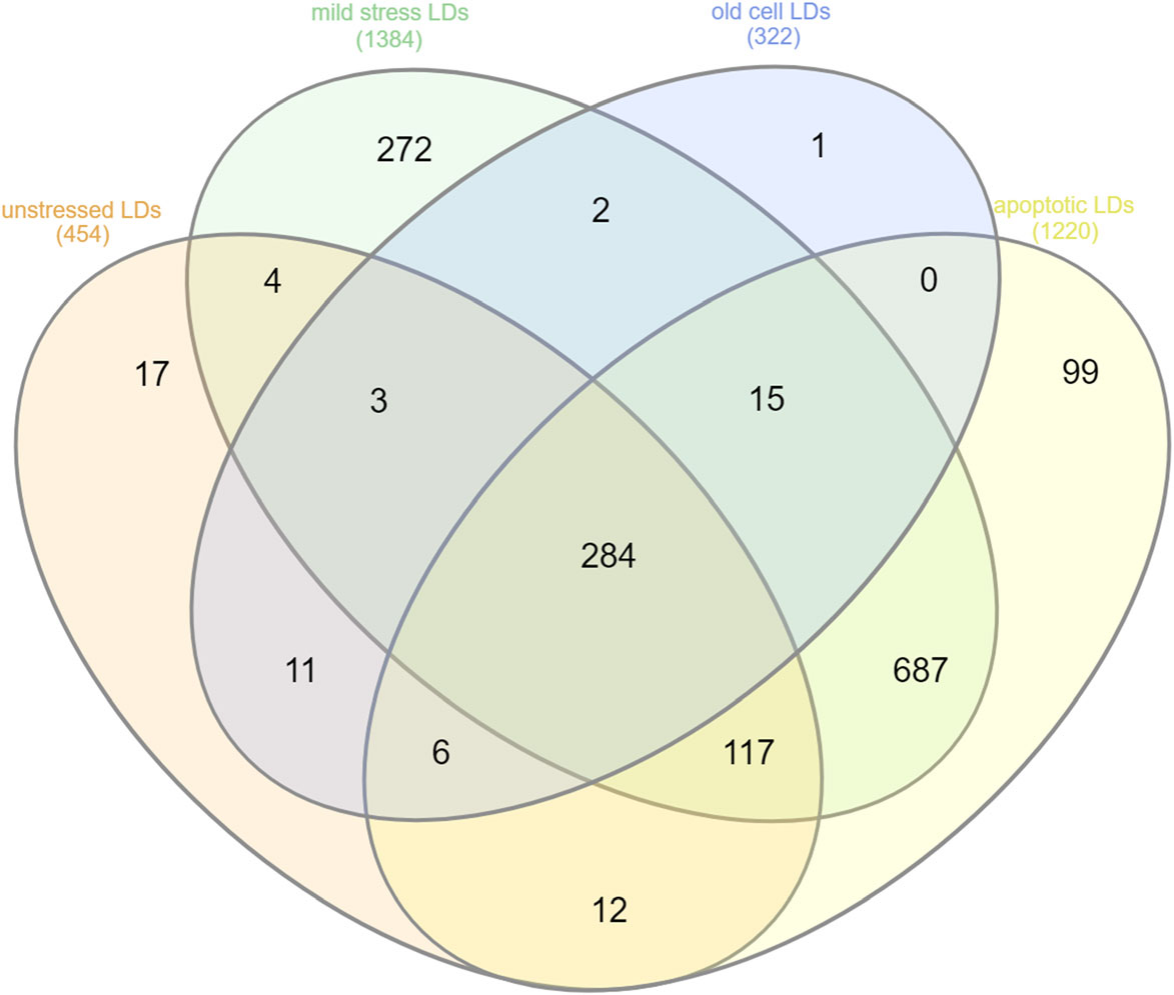
**Venn diagram** comparing the proteome of LDs obtained from unstressed cells (BY4741), stressed cells (BY4741 + 200 mg/L doxycycline), apoptotic cells (BY4741 pCM666-mBAX +200 mg/l doxycycline) and aged cells. Numbers indicate identified proteins. Proteins are listed in supplementary Table 2

In aged cells, the LD proteome increased by only eighteen proteins, of which seventeen were also present at LDs after stress and apoptosis. Only one protein (Gpa1) demonstrated an age dependent LD localization. Gpa1 encodes a subunit of a heterotrimeric G protein and is involved in the yeast mating process. The Gpa1 containing G protein complex activates a phosphatidylinositol 3-kinase in yeast (Slessareva et al. 2006). This is of special relevance as we also demonstrate the increase of phosphatidylinositols in LDs and mitochondria upon aging (see below). Deletion of *GPA1* increases the chronological lifespan of yeast cells (Burtner et al. 2011), and Gpa1 mediated modulation of lifespan seems evolutionarily conserved, as shown in *C. elegans* (Ch'ng et al. 2008). The relocalization of Gpa1 to LDs may provide a mechanism to remove this protein and by such means prolong cellular lifespan. A comparison of the protein numbers in all LD proteomes obtained under different conditions is shown in Fig. 3. A complete protein list of LD specific protein is presented in supplementary Table 1, specific proteins that localize to LDs under various conditions are shown in supplementary Table 2.

### The age and stress proteome of mitochondria

**Fig. 4:**
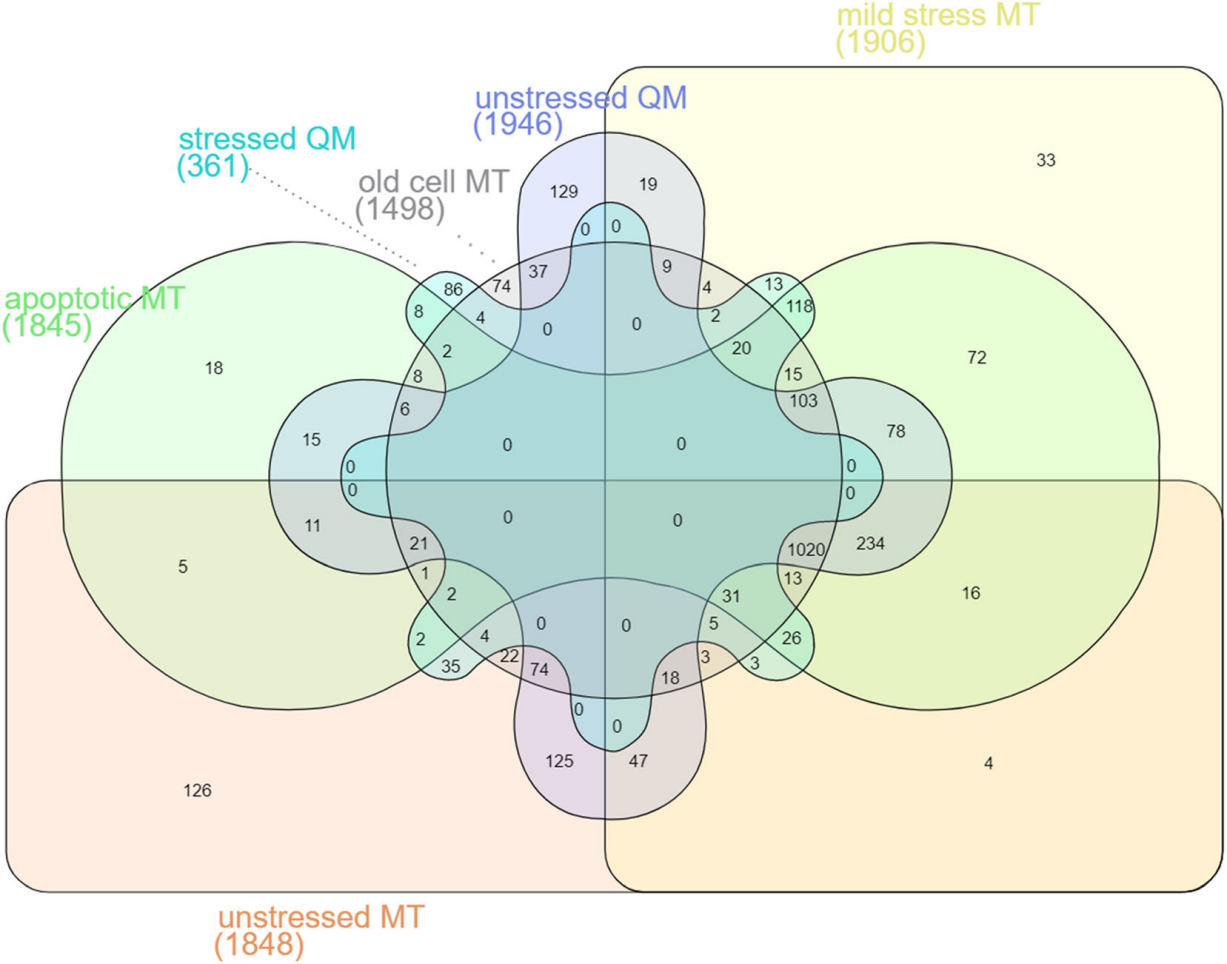
**Venn diagram** comparing the proteome of mitochondria obtained from unstressed cells (BY4741 as well as BY4741 Δare1 Δare2 Δlro1 Δdga1), stressed cells (BY4741 + 200 mg/L doxycycline), apoptotic cells (BY4741 pCM666-mBAX +200 mg/l doxycycline) and aged cells (BY4741 as well as LD deficient BY4741 Δare1 Δare2 Δlro1 Δdga1). Numbers indicate identified proteins. Proteins are listed in supplementary Table 4

The interaction of LDs with organelles including mitochondria, peroxisomes, the nucleus, ER, Golgi apparatus and the lysosome is well documented (Gao and Goodman 2015). We therefore assumed that LDs may act as a protein sink under specific conditions, and clear organelles from harmful proteins and protein aggregates. In this study we focused on the LD-mitochondria interaction. We isolated mitochondria from replicatively aged BY4741 cells, stressed cells (BY4741 + 200 mg/l doxycycline) and apoptotic cells (BY4741 pCM666-mBAX +200 mg/l doxycycline) and from the LD deficient QM cells. The QM strain lacks the ability to synthesize nonpolar lipids and therefore forms no LDs (Bischof et al. 2017). Consequently, this strain background lacks the proposed mitochondrial clearance function via LDs. Mass spectrometric analyses of isolated mitochondria revealed changes in the proteome under all conditions chosen. The change in numbers was less pronounced compared to the observed changes at LDs. We detected 1848 proteins at mitochondria obtained from unstressed cells. Approximately 500 proteins appeared at mitochondria obtained from stressed as well as apoptotic cells (see supplementary Tables 3 and 4). Interestingly, a similar number of proteins was not detectable in stressed mitochondria. Mild and severe stress induced the appearance of proteins with a variance of approximately 10%. The LD deficient QM strain showed a higher number of identified proteins (100 additional proteins). Aged mitochondria presented a 20% decrease in protein number, despite the fact that 269 proteins specifically shuttle to this organelle. We observed an overlap of approximately 50% between apoptotic and stressed cells. We mainly aimed at the identification of proteins that shuttle to mitochondria upon stress and aging and then translocate to LDs. Therefore, we compared the proteins that are specifically present in mitochondria upon mild proteotoxic stress and apoptosis with proteins that appear at LDs after identical conditions. 112 proteins were identified, which followed this pattern (see Table 1). Clustering ((https://david.ncifcrf.gov/)) identified an over-representation of proteins involved in amino-acid biosynthesis (29 proteins), oxidoreductase activity (31 proteins), lyase activity (15 proteins) and ubiquitin like modification pathways (20 proteins). We additionally identified proteins of the ribosome and the proteasome at LDs in stress conditions. A comparison of all mitochondrial proteomes and their overlap is presented in Fig. 4. The corresponding protein lists are presented in supplementary Table 4, whereas the list of all mitochondrial proteins is given in supplementary Table 3.

**Table 1:** : Proteins that show a stress and apoptosis induced translocation to mitochondria followed by a shuttling to LDs

HIS1; HIS4; TRP2; TRP3; ADE4; ARG4; LEU2; MET6; RPS31; MET17; ADE3; GDH1; LYS2; MET3; ARO1; TUB3; RPS21A; ADH4; SAM1; ZWF1; PHO8; INO1; GLN4; HOM2; ARO3; POL30; THR4; MET16; FUR1; PRE10; SCL1; ADE2; PRE1; ARG1; HSP12; PRE8; AGP1; UGA3; URA7; SUA7; TRR1; PMI40; CYS3; PRO3; EUG1; KAP122 ZRT1; NTF2; PGM1; HIS7; SRY1; DOA1; TYS1; GLY1; PDX3; ADH5; ARA1; RIB5; RTC3; ARO9; VTC5; ADE6; LYS9; HXT7; STM1; MET10; DLD3; HMF1; SER3; QDR2; AVT7; SER33; RPL34B; GLR1; AIP1; BNA3; VPS53; BNA1; ADO1; MET5; HLJ1; ASN1; ASN2; IMD3; ARI1; ORM1; MDR1; LSB1; GOR1; ALD3; ERG13; PRO2; RPL34A LAP3; YSA1; MET14; SNO1; SNZ1; OST6; HSP31; GAD1; SVF1; HRI1; ADE13; GRE2; SGT2; YGL117W; YPR148C; YPR097W; YDR391C; YML131W

**Fig. 5:**
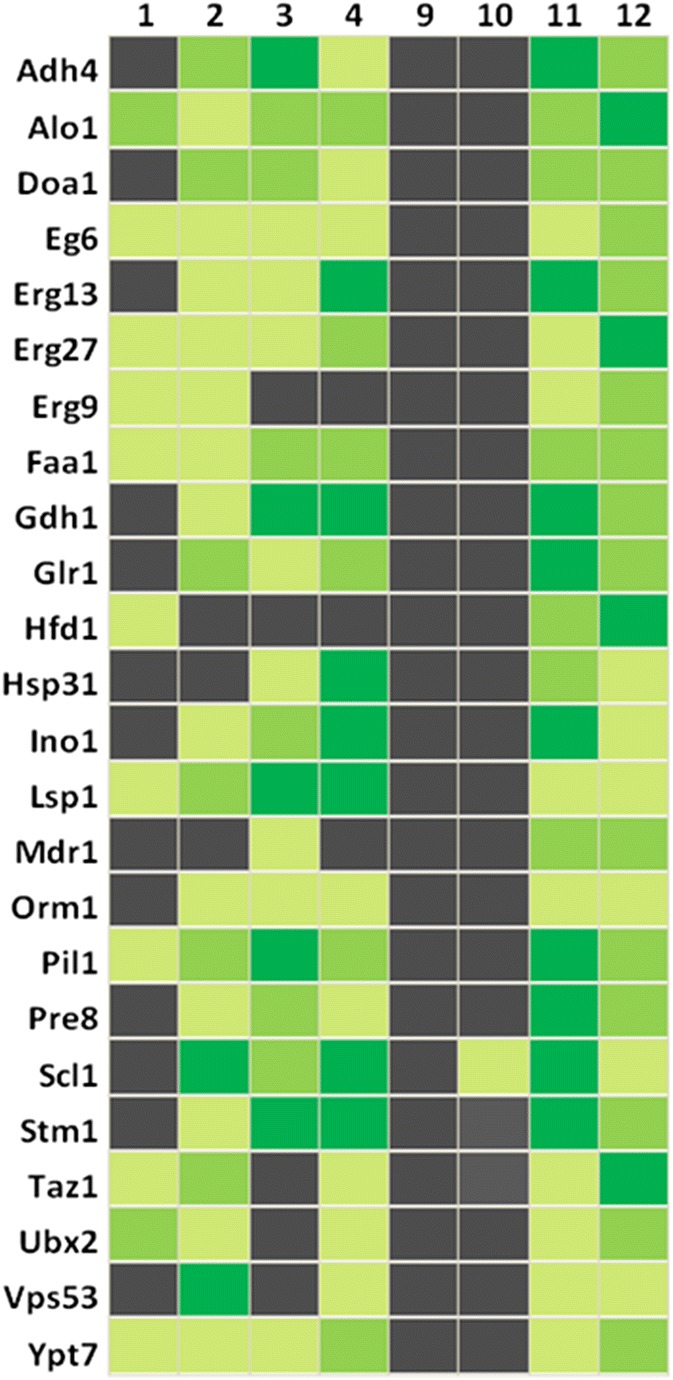
**Heatmap of selected protein localizations.** Black indicates the absence or near absence of proteins. Different shades of green represent various amounts of proteins, from light green (low abundance) to dark green (high abundance). 1: Mitochondria from BY4741; 2: Mitochondria from replicatively aged BY4741 cells; 3: Mitochondria from stressed cells (BY4741 pCM666 + 200 mg/l doxycycline); 4: Mitochondria from apoptotic cells (BY4741 pCM666-mBAX +200 mg/l doxycycline); 9: LDs from BY4741; 10: LDs from replicatively aged BY4741 cells; 11: LDs from stressed cells (BY4741 pCM666 + 200 mg/l doxycycline); 12: LDs from apoptotic cells (BY4741 pCM666-mBAX +200 mg/l doxycycline)

**Fig. 6:**
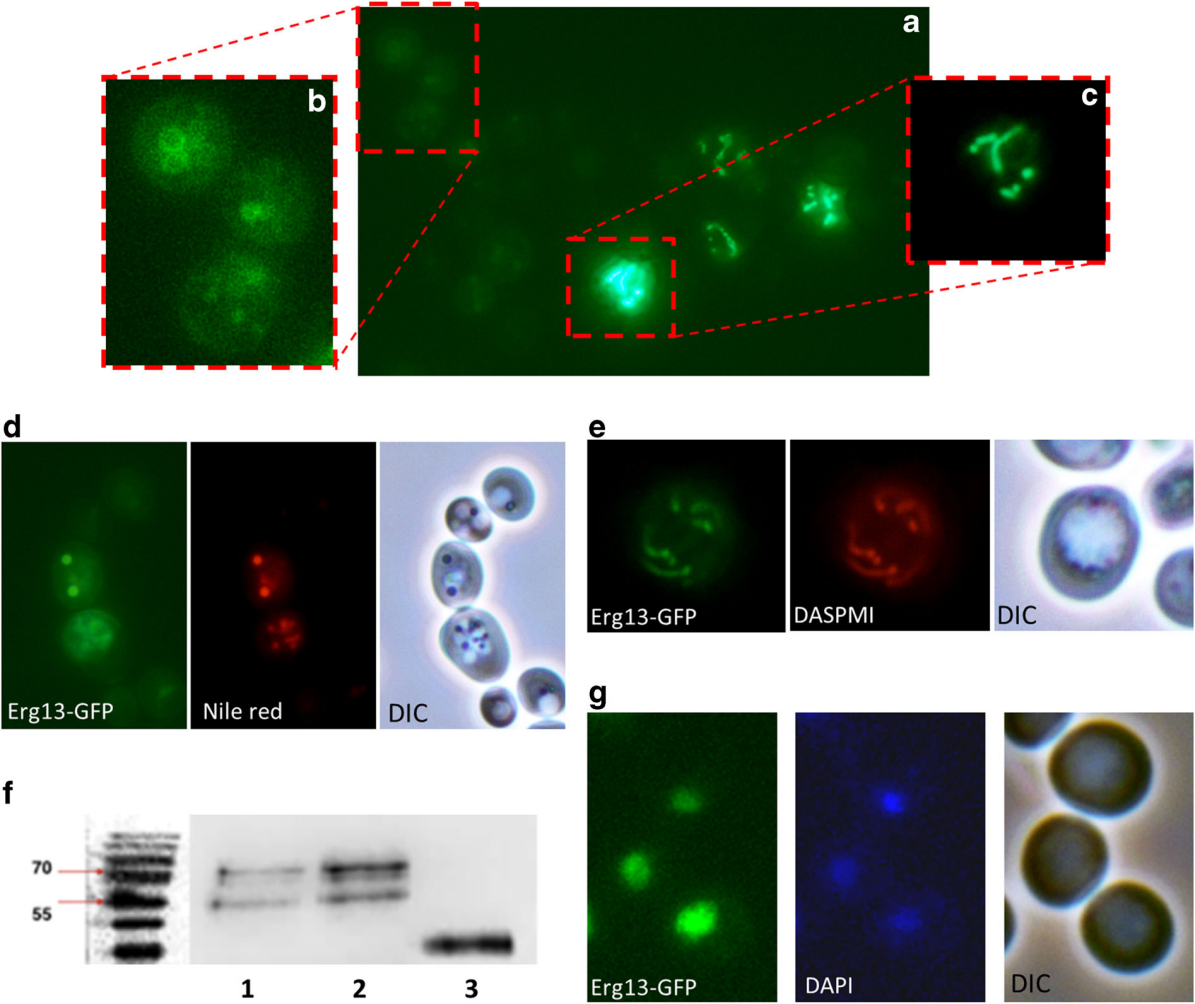
**LD and mitochondrial localization of Erg13.** A) mBAX expressionErg13-GFP localizes either to LDs or mitochondria. A heterogeneous expression level of Erg13-GFP is evident. (B) is a magnification of (A) and shows Erg13-GFP stained LDs. (C) is a magnification of (A) with a reduced exposure time. Erg13-GFP resides at mitochondria. (D) Erg13-GFP co-localizes with LD specific Nile red staining and LDs in the DIC image. (E) The GFP signal of Erg13 completely overlaps with DASPMI (a mitochondrial specific dye). (F) Immunoblot of purified LDs detecting the Erg13-GFP fusion using an anti-GFP antibody. 1: BY4741 ERG13::GFP::HIS3MX6 YEp51 (unstressed cells); 2: BY4741 ERG13::GFP::HIS3MX6 YEp51-mBAX (apoptotic cells); 3: BY4741 pUG35-vBAX (control cells). Erg13-GFP (83 kDa) shows a low abundance at isolated LDs obtained from unstressed cells and shows a high abundance at LDs isolated from apoptotic cells. The positive control vBAX-GFP (33 kDA) confirms the isolation of LDs. (G) Erg13-GFP shows a perfect co-localization with the DNA specific dye DAPI. In the images (A), (B), (C), (D) and (E) the BY4741 ERG13::GFP::HIS3MX6 YEp51-mBAX background is used (apoptotic cells), whereas in (G) the BY4741 ERG13::GFP::HIS3MX6 background (unstressed cells) is shown

The overall picture seems to be more complex, because other conditions may contribute to the presence or absence of particular proteins at either mitochondria or LDs. We found 251 proteins at mitochondria, including Hfd1 and Ubx2, which were absent upon aging and stress. Simultaneously, many of these proteins appeared at LDs. Hfd1 is associated with apoptosis (Manzanares-Estreder et al. 2017), whereas Ubx2 seems to be involved in the process of aging (Labunskyy et al. 2014). Selected proteins and their organelle localization upon cell stress and aging are presented in Fig. 5. Erg13 is of special interest, as it is a nuclear protein and its shuttling to mitochondria upon stress was surprising. Erg13 encodes a 3-hydroxy-3-methylglutaryl-CoA synthase and is involved in ergosterol biosynthesis (it catalyzes the conversion from acetyl-CoA to 3-hydroxy-3-methylglutaryl-CoA). This is in line with our finding that ergosterol levels at mitochondria as well as LDs change dramatically during aging and in stress response (as will be presented later on). Erg13 is also essential for the formation of a tubular mitochondrial network (Altmann and Westermann 2005). Therefore, a strain harboring GFP-tagged *ERG13* (Huh et al. 2003) in the genome was transformed with galactose inducible mBAX or control vector. As shown by fluorescence microscopy and co-staining with DAPI (Fig. 6G) a nuclear localization of Erg13-GFP was observed. Strikingly, induced expression of murine BAX resulted in relocalization of Erg13-GFP to mitochondria and LDs (Fig. 6A, B and C). Fig. 6A also reveals large differences in GFP expression levels. BAX expression induced a modest Erg13-GFP signal in LDs, whereas in mitochondria the GFP signal was significantly higher and even brighter as in the nucleus. We confirmed the presence of Erg13 at LDs and mitochondria by co-staining with LD specific Nile red (Fig. 6D) and mitochondrial specific DASPMI (Fig. 6E). The nuclear localization of Erg13 is fragile and in some clones, the stress of harboring the empty plasmid control was sufficient to induce a mitochondrial and LD localization. We confirmed the observed phenotype by Immunoblot analysis. LDs were isolated from cells with genomically GFP-tagged Erg13, transformed either with YEp51 or YEp51-mBAX. As a positive control we used a two-helical-GFP tagged domain of the BAX protein that we have termed V domain previously. This domain shows a high affinity for LDs (Bischof et al. 2017). By using an anti-GFP antibody we tracked the localization of Erg13-GFP (82 kDA) in the cell. As such, we demonstrated the strong accumulation of Erg13 at LDs upon the expression of BAX (Fig. 6F).

### Stress and aging change the lipidome of LDs and mitochondria

Previously it was demonstrated that LDs and IBs partially colocalize and precede efficient IB clearance. The removal of cytosolic protein aggregates is restricted to a certain subset of LDs. One specific protein at this LD subclass is Pdr16, a phosphatidylinositol transfer protein (Moldavski et al. 2015). Our findings are in line with and support the existence of different LD subtypes. Only a certain LD subclass defined by size had the capacity to accept certain proteins from the OMM in our experiments. Interestingly, we provide evidence that upon apoptosis the number of LDs increases (Bischof et al. 2017), whereas their size decreases (unpublished data). The reduction in LD size upon stress may be a consequence of the detachment of LDs from the endoplasmic reticulum prior to their interaction with other organelles (e.g. mitochondria). With the loss of contact to the ER, LDs seem to lose their constant lipid supply, finally resulting in a decreased size. The LD detachment from the ER and interaction with mitochondria is associated with a change in lipid content. Recent publications also indicate that LDs can act as a store for lipids that are potentially harmful to the cell. Diacylglycerols (DAGs), ceramides (Cers) and acylcarnitines can be transferred to LDs. This way LDs act as a lipid storage compartment to prevent lipotoxicity (Petan et al. 2018). To test these hypothesis we analyzed the lipidome of isolated LDs and mitochondria of the BY4741 (LDs and mitochondria) and QM strain (only mitochondria) with or without the expression of BAX. During differential centrifugation, we carefully avoided cytosolic contaminations in our isolates.

The glycerophospholipid composition of yeast mitochondria is well documented. The mitochondrial membrane consists of 33% phosphatidylcholine (PC), approximately 23% phosphatidylethanolamine (PE), 21% phosphatidylinositol (PI), 3% phosphatidylserine (PS), 7% cardiolipin (CL) and approximately 2% phosphatidic acid (Klug and Daum 2014). We confirmed a similar composition in unstressed cells and observed changes in the lipidome under all conditions tested in the main lipid classes.

**Fig. 7:**
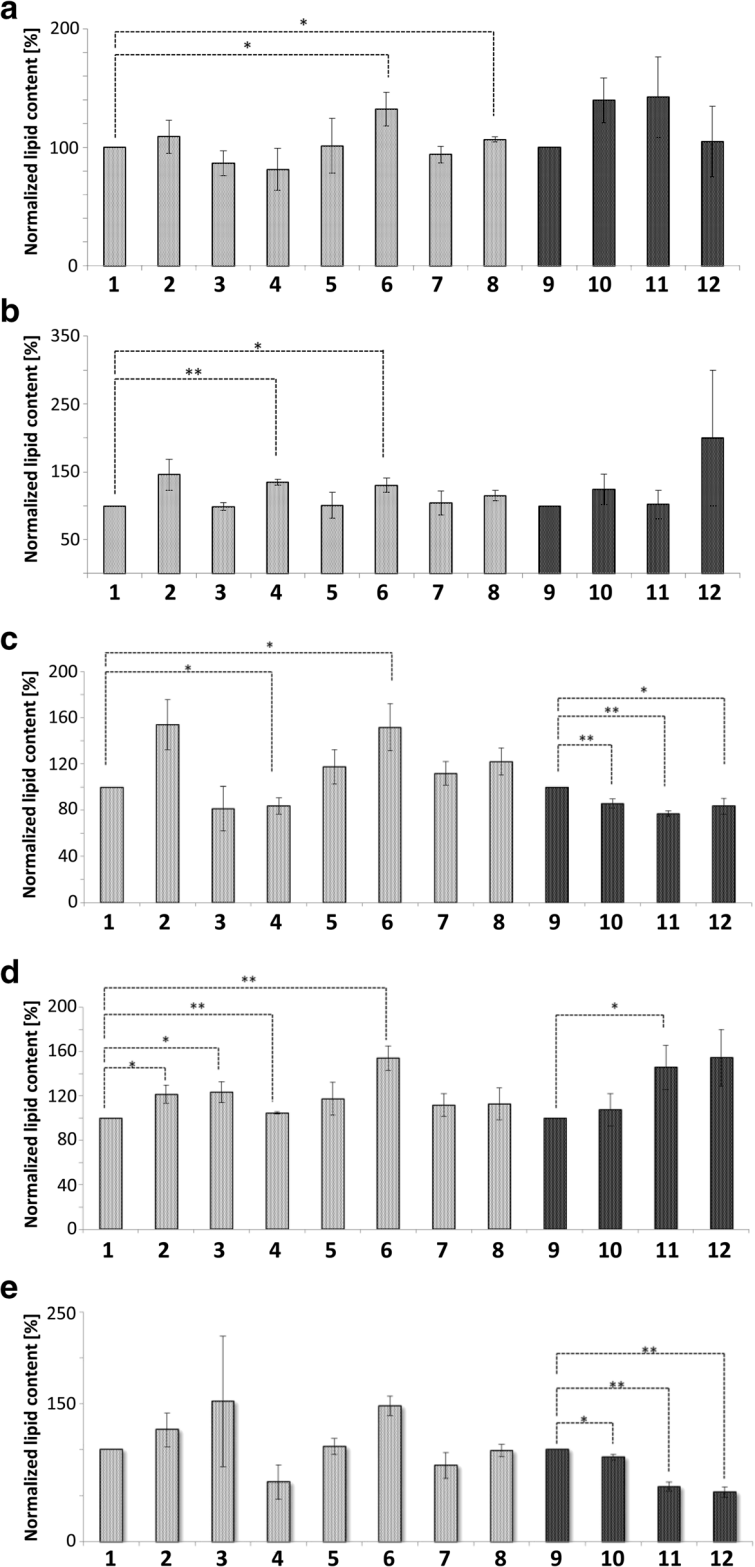
**Glycerophospholipid content of Mitochondria and LDs.** Mitochondria represented in gray, LDs in black. 1: Mitochondria from BY4741; 2: Mitochondria from replicatively aged BY4741 cells; 3: Mitochondria from stressed cells (BY4741 pCM666 + 200 mg/l doxycycline); 4: Mitochondria from apoptotic cells (BY4741 pCM666-mBAX +200 mg/l doxycycline); 5: Mitochondria from the strain BY4741 *Δare1 Δ are2 Δlro1 Δdga1*; 6: Mitochondria from the replicatively aged strain BY4741 *Δare1 Δ are2 Δlro1 Δdga1*; 7: Mitochondria from the stressed strain BY4741 *Δare1 Δ are2 Δlro1 Δdga1* pCM666 + 200 mg/l doxycycline*;* 8: Mitochondria from the apoptotic strain BY4741 *Δare1 Δ are2 Δlro1 Δdga1* pCM666-mBAX +200 mg/l doxycycline; 9: LDs from BY4741; 10: LDs from replicatively aged BY4741 cells; 11: LDs from stressed cells (BY4741 pCM666 + 200 mg/l doxycycline); 12: LDs from apoptotic cells (BY4741 pCM666-mBAX +200 mg/l doxycycline). In (A) phosphatidylcholines, in (B) phosphatidylethanolamines, in (C) phosphatidylinositols and in (D) phosphatidylglycerols

*Phosphatidylethanolamine*: Besides PC, PE is the most abundant phospholipid in membrane bilayers (ranging from 20%–50%) (Schuiki et al. 2010). PC together with PS (both have a cylindrical shape) are required to form flat layers. In contrast to the above mentioned lipid sub-classes, PEs are cone-shaped and therefore responsible to “bend” layers leading to a curvature of the membranes. Our analysis revealed the increase of PEs under all conditions tested (Fig. 7B). Aged cells showed a 1.5-fold increase in PE content in mitochondria and LDs. Apoptotic cells displayed a 1.5-fold increase of PEs in mitochondria and a 2-fold increase in LDs. The stress- and age-induced change in PE content may be a consequence of several physiological interactions. Upon stress and increased age, the mitochondrial tubular network starts to fragment (Klinger et al. 2010), and LDs show a reduced size. The transformation of the mitochondrial network to “dotted” structures as well as the reduction of LD size could be supported by increased PE contents that promote membrane curvature. Furthermore, PEs can flip-flop from the cytosolic to the exoplasmic leaflet in the plasma membrane and this way contribute to apoptosis. Recently, it was demonstrated that an increase in PE content of membranes is a stimulus for autophagy (Rockenfeller et al. 2015). By such a mechanism, the observed increase in mitochondrial PE could support the elimination of damaged mitochondria by mitophagy. The increased content of PE in LDs may assist in elevating lipophagy rates and therefore causing the elimination of LDs harboring toxic lipids and proteins. Additionally, the lipophagic degradation of LDs refuels the cell with energy needed for regrowth after times of stress.

**Fig. 8:**
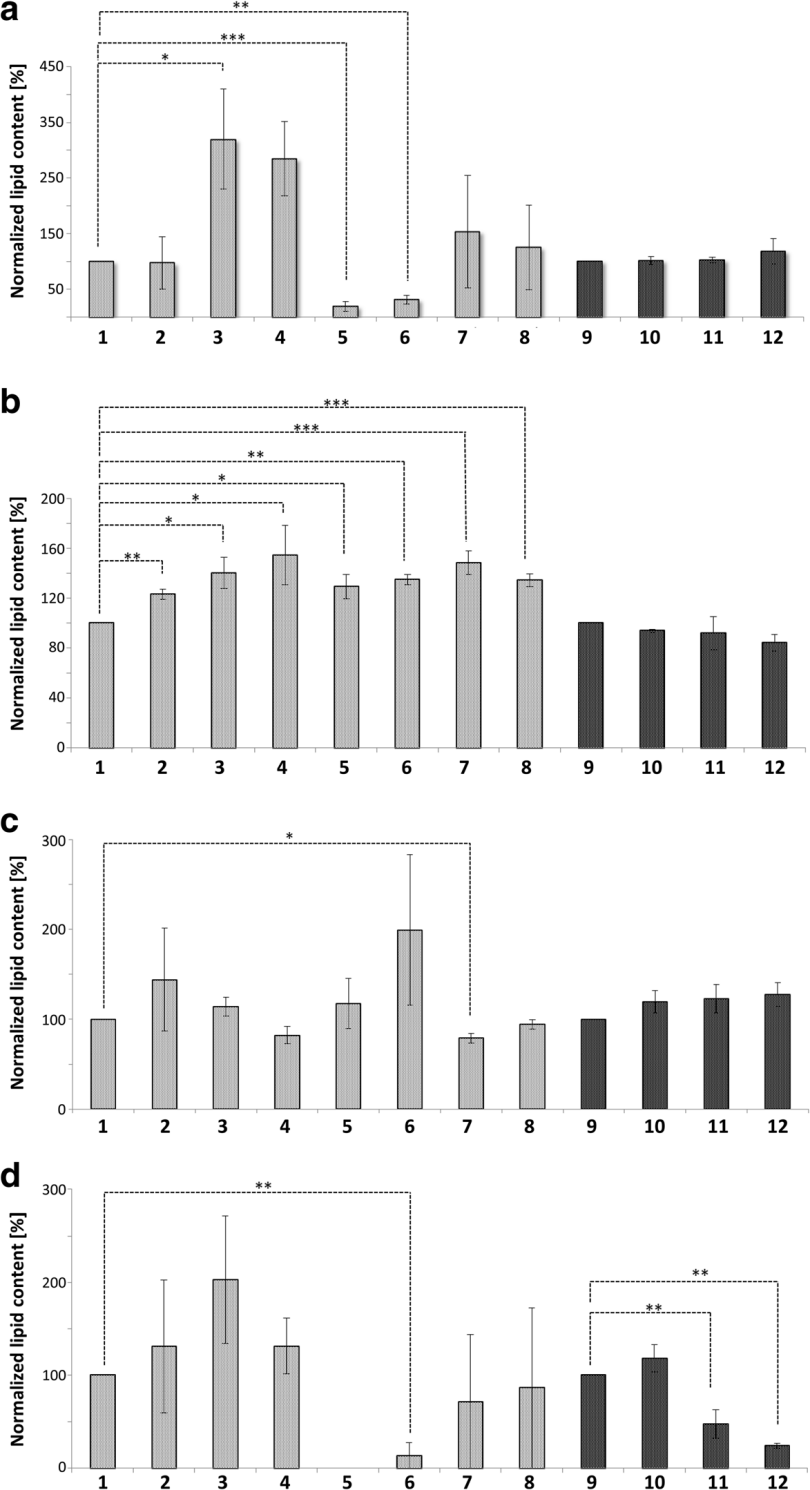
**Lipid content of Mitochondria and LDs.** Mitochondria represented in gray, LDs in black. 1: Mitochondria from BY4741; 2: Mitochondria from replicatively aged BY4741 cells; 3: Mitochondria from stressed cells (BY4741 pCM666 + 200 mg/l doxycycline); 4: Mitochondria from apoptotic cells (BY4741 pCM666-mBAX +200 mg/l doxycycline); 5: Mitochondria from the strain BY4741 *Δare1 Δ are2 Δlro1 Δdga1*; 6: Mitochondria from the replicatively aged strain BY4741 *Δare1 Δ are2 Δlro1 Δdga1*; 7: Mitochondria from the stressed strain BY4741 *Δare1 Δ are2 Δlro1 Δdga1* pCM666 + 200 mg/l doxycycline*;* 8: Mitochondria from the apoptotic strain BY4741 *Δare1 Δ are2 Δlro1 Δdga1* pCM666-mBAX +200 mg/l doxycycline; 9: LDs from BY4741; 10: LDs from replicatively aged BY4741 cells; 11: LDs from stressed cells (BY4741 pCM666 + 200 mg/l doxycycline); 12: Mitochondria from apoptotic cells (BY4741 pCM666-mBAX +200 mg/l doxycycline). In (A) triacylglycerols, in (B) ceramides, in (C) phosphatidic acids and in (D) ergosterols are presented

*Ergosterol*: Yeast cells differ from mammalian cells as they use ergosterol as their main sterol. The fungi modulate membrane permeability and fluidity by alterations in ergosterol content. Cells may synthesize ergosterol de novo, or take it up from the environment. The latter case is essential under anaerobic conditions and main storage sites for esterified ergosterols are LDs (Jacquier and Schneiter 2012; Rodrigues 2018). Under stress conditions, ergosterol is inversely distributed between LDs and mitochondria (Fig. 8D). Mild stress reduced the ergosterol content in LDs by 50% and in “apoptotic” LDs by 70%. In contrast, mitochondrial ergosterol content increased in stressed/apoptotic cells between 1.5 to 2-fold. The involvement of ergosterol in stress response is plausible, as yeast mutants defective for ergosterol biosynthesis are sensitive to oxidative stress (Thorpe et al. 2004). Strikingly, accumulation of ergosterol in the membranes increases stress resistance, probably caused by the decreased permeability for hydrogen peroxide. Therefore it seems reasonable that LDs supply mitochondria with ergosterol (Branco et al. 2004). By this mechanism, damaged mitochondrial membranes can retain hydrogen peroxide more effectively, thus limiting the cellular damage. Our experiments demonstrate that cells devoid of LDs do not contain detectable ergosterol in mitochondria (Fig. 8D). During aging as well as after stress application we found increased biosynthesis of ergosterol and its incorporation into mitochondrial membranes in wildtype cells. This indicates an important role of ergosterol during the aging process and stress response. It is also important to notice that many components of the ergosterol biosynthesis pathway such as Erg6, Erg13, Erg27 and Erg9 change their cellular location upon aging and during the stress response (Fig. 5 and Fig. 6).

*Triacylglycerols:* Free fatty acids (FFAs) are stored as neutral lipids in the form of triacylglycerols in LDs. Most components of the TAG biosynthetic machinery in yeast reside in the ER and in LDs, although a mitochondrial acyltransferase (Taz1) exists (Eisenberg and Buttner 2014). Our results demonstrate the increase of Taz1 in mitochondria upon aging and in LDs of stressed and apoptotic cells (Fig. 5). TAGs are constantly synthesized and degraded throughout the entire cell cycle. The breakdown of TAGs is catalyzed either by LD-resident lipases or by lipophagy (Walch et al. 2015). The resulting oxidation of fatty acids supplies the cell with energy. A trigger for TAG breakdown is the G1/S transition, as cells require energy to initiate DNA replication and to form buds. TAGs accumulate in mitochondria during mild stress and apoptosis. Two reasons may explain this finding: (1) Stress is accompanied with cell cycle arrest and low demand for energy, which changes as soon as the cells start to regrow. (2) FFAs can promote cell death, as they increase ROS production in times of stress thus leading to a vicious circle (Eisenberg and Buttner 2014; Kohlwein 2010; Li et al. 2018; Schonfeld and Wojtczak 2008). Storing FFAs as TAGs significantly reduces lipotoxicity. The increased amount of TAGs in mitochondria (Fig. 8A) may be regarded as a potential protection mechanism. Unstressed LD deficient mutant cells have low mitochondrial TAG levels, which increase significantly upon stress and apoptosis.

*Phosphatidylinositol (PI)*: PIs have multiple roles in the cell and are present in cellular membranes. Several hydroxyl groups can be phosphorylated thus generating phosphoinositides, which are enzymatically cleaved and generate second messengers such as DAG and IP3 (inositol-1,4,5-P_3_) (Strahl et al. 2005). Many pathways utilize this mechanism and targets for these second messengers are manifold (e.g. the cytoskeleton, cellular trafficking and gene transcription) (Guillas et al. 2013). In addition, PIs contribute to autophagy, in which an early hallmark consists in the formation of PIP3 by PI3K. This phosphoinositide was found to accumulate at autophagosomes (Cebollero et al. 2012). In fact, we observed increased PI levels in aged mitochondria, which possibly indicate an increased rate of autophagy (Fig. 7C). In line with this is our observation that PI levels decrease in LDs derived from aged as well as stressed/apoptotic cells. This indicates that the LDs act as the donor for mitochondrial PIs. On the other hand, we observed increased PIs in aged mitochondria in the LD deficient mutant strain background. As increased PI levels are specific for aged mitochondria, we speculate about a role of PIs at mitochondria in the aging process independent of autophagy. In this context, it is of interest that stress induced a relocalization of Sac1. Sac1 dephosphorylates phosphoinositides and predominantly resides in the ER, but a mitochondrial localization was reported too (Strahl and Thorner 2007). We show an apoptosis and stress induced localization of Sac1 specifically at LDs for the first time.

*Phosphatidic acid*: PAs are key metabolites in many cellular pathways of lipid biosynthesis, and phospholipids as well as TAGs are formed from phosphatidic acid (Klug and Daum 2014). We observed a replicative age dependent increase of PAs in mitochondria, similar to PIs (Fig. 8C). This is in concordance with the dataset of Titorenko and colleagues reporting the increase of PAs in mitochondria isolated from chronologically aged yeast cells (Burstein and Titorenko 2014; Leonov et al. 2017). In addition, decreased levels of PE and cardiolipin, but increased levels of PS, PG and PC were found (Burstein and Titorenko 2014). Altered mitochondrial PA levels are most probably attributed to age dependent changes of mitochondrial functions as well as fragmentation of the mitochondrial network. In addition, it was demonstrated that differences in PA levels have an impact on mitochondrial function and morphology (Mesmin 2016).

In contrast to mitochondria, we detected increased PA levels in LDs isolated from aged as well as stressed/apoptotic cells. PAs have the capability to modulate LD size (Fei et al. 2011a). We suggest that the correct size of LDs is crucial for the formation of inter-organelle contact sites. In line with this, we and others demonstrated that only certain size-dependent subtypes of LDs are capable to act as a protein sink (Bischof et al. 2017; Moldavski et al. 2015). Stress as well as most probably aging induces the detachment of LDs from the ER. Due to the loss of ER-mediated lipid supply, the LDs start to shrink. The incorporation of PAs may therefore constitute a mechanism to increase LD size for appropriate interaction with mitochondria.

*Phosphatidylglycerol (PG) and Cardiolipin (CL)*: In contrast to other glycerophospholipids, the relative amount of PG inside yeast cells is only a few tenths of a percent. Based on the low abundance, it is considered to represent mainly a metabolic precursor for CL synthesis (Simockova et al., 2008). CL is involved in different cellular functions including cell growth and the maintenance of mitochondrial biogenesis and function (Pokorna et al. 2016; Simockova et al. 2008). In mitochondria, CL is a prerequisite for correct anchoring of the OXPHOS supercomplex. Furthermore, it participates in the establishment of cristae and contributes to protein import. In line with our proposed model is the role of CL in apoptosis and its capability to mediate membrane fusions and the formation of local non bilayer structures (Joshi et al. 2009; Pokorna et al. 2016). Local non bilayer structures are present at detachment sites of LDs that are still bound to the ER. It supports the concept that ER-bound LDs are in contact via a lipid bridge (Bohnert 2018). We observed a 1.2 fold increase of PG in mitochondria of mildly stressed and replicatively aged cells. No change in PGs was detected in mitochondria of BAX stressed cells (Fig. 7D). This is in line with the concept of PGs as metabolic intermediates, which are constantly used for CL formation. We speculate that CL could support a lipidic bridge/membrane fusion between LDs and mitochondria during stress. This mechanism may assist in improved clearing of the OMM from potential “harmful “proteins (Bischof et al. 2017). Mitochondria from replicatively aged LD deficient mutant cells contained 1.5 fold increased PG levels. This may represent a protection mechanism of a strain deficient in producing LDs. PG can be converted into CL, and we speculate that binding of CL to cytochrome c may interfere with the initiation of the apoptotic program. Decreased CL levels during aging and oxidative stress are often associated with an increased rate of apoptotic cell death (Joshi et al. 2009).

Murine BAX expression represents a massive stressor for the yeast cell. In apoptotic cells, the amount of PG in mitochondria was not different from unstressed cells. We assume that the conversion of PG to CL assists in the induction of membrane fusions and quenching of cytosolic cytochrome c, released through Bax-oligomeric pores at mitochondria. An interaction between CL and cytochrome c and its interplay was already demonstrated (Kagan et al. 2009). PGs increased 1.45 fold in LDs of mildly stressed cells, indicating a role in a potential “feeding“-mechanism for mitochondria to increase the amount of CL. This has a protective function during times of elevated stress.

*Ceramide (Cer)*: Cers resemble the core structure and are precursors of sphingolipids (Montefusco et al. 2013). Additionally, yeast cells use Cers as substrates to synthesize complex lipids, which constitute about 10% of all membrane lipids (Cowart and Obeid 2007). Cers have a smaller head size than sphingomyelins, enabling the induction of asymmetric membrane tension. As an outcome, the tendency to form highly ordered domains of high shear viscosity ensues (Lopez-Montero et al. 2007), and thus Cers can perturbate membrane structures. Various ceramides have different effects on cells, some of which are toxic whereas others are protective (Epstein et al., 2012). Cers participate in a variety of cellular processes, including oxidative stress, apoptosis and can act as second messengers (Lopez-Montero et al. 2007). Elevated Cer levels have multiple effects: A particular subform of apoptosis, as well as cell growth arrest are initiated (Eisenberg and Buttner 2014). Specific ceramides perturb membranes, a mechanism involved in the initiation of apoptosis, whereas a reduction in ceramide levels is associated with cellular growth arrest (Eisenberg and Buttner 2014). Elevated Cer levels contribute to the formation of ROS by direct inhibition of mitochondrial complex III of the respiratory chain. Furthermore Cers cause morphological alterations such as organelle fragmentation and aggregation (Carmona-Gutierrez et al. 2011; Rego et al. 2012). Cers also contribute to the formation of channels in the mitochondrial membrane. The process is regulated by pro-apoptotic proteins of the Bcl2-family and finally results in cytochrome c release and the activation of the apoptotic program. In contrast, various anti-apoptotic proteins are responsible for the disassembling of Cer channels (Ganesan et al. 2010). Our lipid data are in line with these findings (Fig. 8B). We observed a 1.2 fold increase in Cer content of replicatively aged mitochondria in the wildtype strain. The application of mild stress increased Cer levels 1.4 fold; whereas Bax-mediated severe stress increased Cer levels 1.5 fold. We observed a similar pattern of stepwise alteration in mitochondria of the LD deficient mutant strain and 1.3 fold increased Cer content at mitochondria in unstressed as well as replicative aged cells compared to the wildtype. Cer levels increased 1.5 and 1.3 fold under mild and severe Bax-induced stress conditions, respectively. Replicatively aged as well as BAX stressed cells presented only a weak, statistically not significant reduction in the Cer content of LDs (Fig. 8B). Increasing mitochondrial Cer levels were correlated to the intensity of the applied stress. This seems reasonable, as both, Cers and BAX participate in the induction of apoptosis. ER resident Orm1 specifically appeared in LDs and mitochondria and may contribute to the age and stress induced mitochondrial increase of Cers and the simultaneous decrease in LDs. Orm1 is involved in sphingolipid homeostasis and its deletion increased sphingolipid levels as well as sensitivity to stress (Han et al. 2010). Another example for our observed connection of the proteome with the lipidome is Hfd1. During apoptosis Hfd1 accumulates at the OMM and is involved in the degradation of sphingolipids. The sphingolipid degradation product hexadecenal induces cytoskeletal reorganization and apoptosis in a JNK-dependent manner and regulates mitochondrial function. High levels of hexadecenal trigger the fragmentation of the mitochondrial network and are accompanied by increased ROS levels (Manzanares-Estreder et al. 2017). We confirmed this mechanism with our results. Unstressed cells contain small amounts of Hfd1p at mitochondria, but levels increase from mildly to severely stressed cells. The accumulation of Hfd1 at LDs under stress conditions may assist in the reduction of toxic hexadecenal and prevent mitochondria from further damage.

## Conclusion

In this work we focused on the interaction of LDs with mitochondria and the specific transfer of proteins and lipids from mitochondria to LDs. We characterized the proteome and lipidome of stressed, apoptotic and aged cells. Changes in protein composition are depending on the applied stress. Mild proteotoxic stress and apoptosis induced dramatic changes in the protein profile, whereas in aged cells the changes were only modest.

Among the proteins that emerge at mitochondria and are redirected to LDs in stressed cells, we found an enrichment of proteins with oxidoreductase activity and participation in amino-acid biosynthesis. During aging, we identified several “shuttling” proteins that regulate lipid metabolism, the metabolism of distinct fatty acids as well as oxidative stress response. The stress and age proteome of LDs associated with the ergosterol biosynthesis pathway (Fig. 5). Erg27 contributes to ergosterol biosynthesis, and was low-abundant in mitochondria of unstressed, mildly stressed and replicatively aged cells. In apoptotic cells Erg27 accumulated in mitochondria (approximately 4-fold increase). We observed a similar pattern in LDs, as Erg27 was nearly undetectable under unstressed conditions. In dependence of the applied stress Erg27 was present at LDs. In apoptotic cells, the amount of Erg27 was more than 13 fold increased compared to wildtype. This is in line with a potential transfer of Erg27 from mitochondria to LDs under stress conditions. Although less pronounced, we observed an identical pattern for Erg6 and Erg9. A further example is Erg13, for which we additionally confirmed the relocalization from the nucleus to mitochondria as well as LDs by fluorescence microscopy (Fig. 6). The fluorescence images also confirmed highly increased Erg13 expression (mass spectrometry data indicate a more than 59-fold increase in Erg13 levels at LDs). Our finding may be indicative for increased cellular demand for ergosterol under stress. One reason could be that increased amounts of ergosterols in mitochondria of mildly stressed as well as apoptotic cells can decrease membrane permeability for ROS.

Besides a beneficial effect, highly abundant ergosterol levels may interfere with cellular integrity by increasing membrane stiffness. Overexpression of all enzymes of the ergosterol biosynthesis pathway (including Erg6, Erg9, Erg13 and Erg27) were harmful to yeast cells (Bhattacharya et al. 2018). Therefore, enzymes of this pathway need specific fine-tuning in expression to assure cell survival under unfavorable conditions. Modulation of cell membrane composition also seems to have a positive effect on longevity as demonstrated in several organisms (Delhaye et al. 2016).

The shuttling of these enzymes may assist in such a fine-tuning process. Ergosterol biosynthesis strongly increased in stressed and aging cells and LDs supply the mitochondria with this lipid. Consequently, we report significantly reduced ergosterol levels at LDs from mildly stressed and apoptotic cells (Fig. 8D).

The observed changes in the proteome reflect corresponding alterations in the lipidome. In general, we observed increased levels of lipids in mitochondria and LDs under all conditions tested (stress, apoptosis and aging). There is growing evidence that lipotoxicity contributes to biological aging in general (Carter et al. 2019). Increased PGs serve as building blocks for CL synthesis and elevated mitochondrial CL may decrease the release of cytochrome c. This indicates that elevated levels of PG have anti-apoptotic functions under these conditions. In contrast, mitochondrial Cer levels were positively associated with the severity of the applied stress. Cers display clear pro-apoptotic effects and may induce pore formation and cytochrome c release in mitochondria. The additive effect of Cers and BAX may irreversibly stimulate apoptosis. In the case of PE higher amounts in mitochondria are necessary for a higher curvature of membranes and thus a transition from the tubular network to the fragmented network that is typical for aging and apoptosis. It is plausible that PEs and PAs in LDs fulfill a similar role and contribute to organelle size adaption, a prerequisite for contact with mitochondria. Direct contact seems essential to redirect specific proteins from mitochondria to LDs. Therefore, fine-tuning of the lipidome and proteome is necessary to increase cell survival upon stress and aging. Our data support the notion that LDs are key players in this important balancing act. We regard LDs as vehicles with the ability to clear other organelles from potentially dangerous proteins and lipids by sequestering them into the vacuole. Via this process of lipophagy, a specialized kind of autophagy, LDs have direct influence on aging and longevity (Hansen et al. 2018).

## Electronic supplementary material

Supplementary table 1 Complete list of all LD proteins from healthy WT cells, stressed WT cells, apoptotic WT cells and aged WT cells. (XLSX 105 kb)

Supplementary table 2 List of LD proteins specific for selected conditions (Fig. 3) (XLSX 1066 kb)

Supplementary table 3 Complete list of all mitochondrial proteins from WT cells, stressed WT cells, apoptotic WT cells, aged cells, QM cells, stressed QM cells, apoptotic QM cells and aged QM cells. (XLSX 102 kb)

Supplementary table 4 List of mitochondrial proteins specific for selected conditions (Fig. 4) (XLSX 339 kb)
